# Peritoneal Hydatid Cyst Mimicking Peritoneal Seeding; A Case Report

**DOI:** 10.30699/ijp.2025.2056341.3430

**Published:** 2025-07-01

**Authors:** Nasser Malekpour Alamdari, Iman Ansari, Maede Karimian, Elnaz Babakhani, Sara Hatami, Parisa Mohammadsadeghi, Maryam Abbasi

**Affiliations:** 1 *Critical Care Quality Improvement Research Center, Shahid Modarres Hospital, Shahid Beheshti University of Medical Sciences, Tehran, Iran*; 2 *Department of General Surgery, Shahid Modarres Hospital, Shahid Beheshti University of Medical Sciences, Tehran, Iran*; 3 *Department of Anesthesiology, Shahid Modarres Hospital, Shahid Beheshti University of Medical Sciences, Tehran, Iran*; 4 *Department of Pediatrics, Mofid Children’s Hospital, Shahid Beheshti University of Medical Sciences, Tehran, Iran*; 5 *Department of Pathology, Tehran University of Medical Sciences, Tehran, Iran*

**Keywords:** Hydatid Cyst, Echinococcosis, Peritoneal Neoplasms, Neoplasm Seeding, Spleen

## Abstract

**Background & Objective:**

This study presents a rare case of hydatid cysts involving the liver, spleen, and peritoneum, in which clinical features and radiologic findings initially raised strong suspicion for abdominal malignancy with peritoneal seeding.

**Case Presentation:**

A 64-year-old man presented with vague epigastric pain, weight loss, and iron deficiency anemia. Abdominal ultrasonography revealed multiple splenic lesions suggestive of hydatid cysts. Hydatid serology was negative. Chest CT was unremarkable. Abdominal and pelvic CT showed splenomegaly with cystic lesions, including a calcified cyst in segment VI of the liver. Numerous hypodense peritoneal nodules were identified, particularly in the mid-omentum and supraumbilical region. Differential diagnoses included hydatid disease, primary peritoneal neoplasms, and peritoneal metastases. Due to anemia, weight loss, and the suspicion of peritoneal carcinomatosis, hydatid disease alone could not fully account for the findings, prompting further diagnostic evaluation. Endoscopy and colonoscopy were unremarkable. The patient underwent exploratory laparotomy and splenectomy, along with partial omentectomy where hydatid involvement was suspected. Postoperatively, he was treated with albendazole 800 mg daily and discharged on postoperative day three. At 3- and 6-month follow-ups, the patient reported resolution of abdominal pain, and physical examinations were normal.

**Conclusion:**

Peritoneal hydatid disease is rare and can mimic peritoneal carcinomatosis, leading to diagnostic uncertainty and treatment delay. Surgical excision followed by antiparasitic therapy remains the cornerstone of management.

## Introduction

Echinococcosis is a zoonotic parasitic infection caused by tapeworms of the genus *Echinococcus*. Humans act as accidental intermediate hosts and become infected through ingestion of parasite eggs, either via direct contact with infected animals or indirectly through contaminated food or water. According to the World Health Organization, echinococcosis is classified as a neglected tropical disease due to its continued high prevalence in endemic regions despite improvements in public health and sanitation practices (1,2).

The disease is frequently asymptomatic and often detected incidentally during imaging studies for unrelated conditions (3). In most cases, a single cyst is present, and the formation of multiple cysts involving multiple organs is uncommon. The liver is the most frequently affected organ, followed by the lungs. Involvement of other organs—such as the spleen, peritoneum, or omentum—is considerably rarer. When peritoneal or omental involvement occurs, it is typically secondary to abdominal trauma, spontaneous or iatrogenic rupture of a hepatic or splenic cyst, or surgical manipulation, leading to dissemination of protoscolices within the peritoneal cavity (4).

This report presents a rare case of hydatid disease involving the liver, spleen, and peritoneum, in which the clinical presentation and radiologic findings closely mimicked intra-abdominal malignancy with peritoneal carcinomatosis. In addition to describing this case, we provide a brief overview of the epidemiology, clinical features, diagnostic approach, and therapeutic strategies for hydatid disease. A preprint version of this report has been previously published (5).

## Cases Report

A 64-year-old male farmer with elementary education, residing in a rural area of Zanjan province, Iran, presented to our surgical clinic nine months ago with complaints of vague abdominal pain predominantly in the epigastric region. Over this period, he had experienced unintentional weight loss exceeding 10 kg. In the preceding month, he reported additional symptoms including anorexia, generalized weakness, and lightheadedness. The abdominal pain was persistent and unrelated to meals, physical activity, or body position. He denied nausea or any change in bowel habits.

The patient had a five-year history of type 2 diabetes mellitus, managed with metformin 1 gram daily. He had no prior history of surgery, alcohol or tobacco use, or substance abuse, and reported no known food or drug allergies. Family history was notable for a brother with colorectal cancer; no other significant familial illnesses were reported.

On examination, the patient’s vital signs and general physical appearance were within normal limits. Abdominal examination was unremarkable, and no pathological findings were noted on systemic physical evaluation.

Given his weight loss, anemia, and gastrointestinal symptoms, there was an initial clinical suspicion of gastrointestinal malignancy. Laboratory tests revealed microcytic anemia with low hemoglobin, mean corpuscular volume (MCV), serum iron, and ferritin levels. Fasting blood sugar and HbA1c were elevated. Other laboratory values were unremarkable.

**Figure 1:**
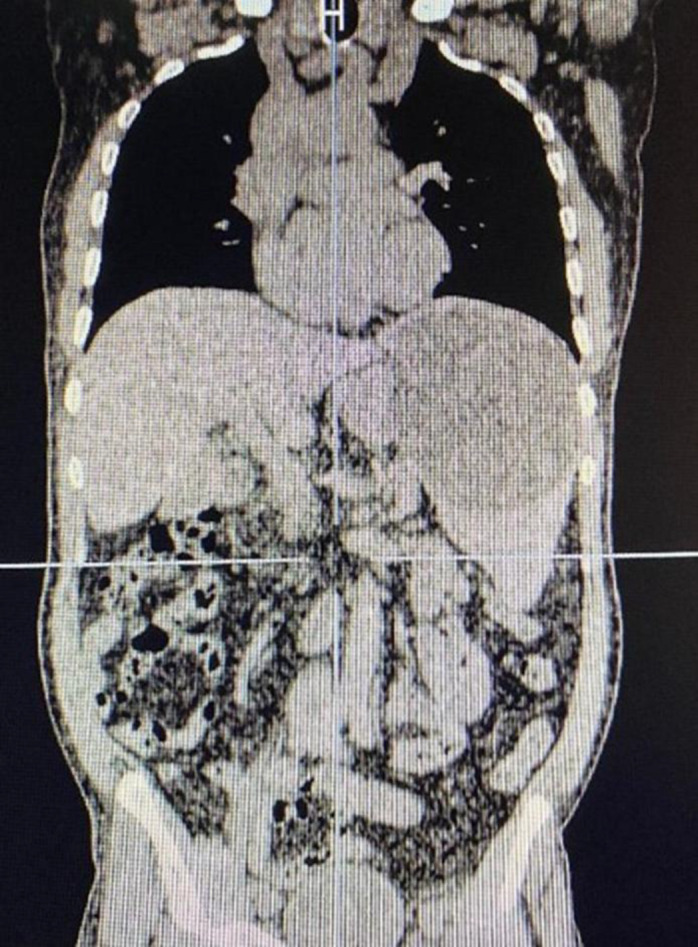
Based on the CT scan of the abdomen and pelvis, there were a large number of peritoneal nodules in different regions of the abdomen and in the middle part of the omentum and above the umbilicus, for which hydatid cysts, primary neoplastic or metastatic lesions to the peritoneum were included in the differential diagnosis.

Abdominal ultrasonography showed a cystic lesion in the liver, splenomegaly, and multiple splenic cysts suggestive of hydatid disease. However, serologic testing for hydatid cyst was negative. A chest CT scan revealed no abnormalities. Abdominopelvic CT showed splenomegaly (anteroposterior diameter: 174 mm), at least six splenic cystic lesions (largest measuring 92 × 108 mm), and one calcified cyst in hepatic segment VI (23 × 33 × 43 mm). Additionally, numerous peritoneal nodules were identified throughout the abdomen (largest measuring 48 × 27 mm), along with six hypodense structures in the mid-omentum and supraumbilical region (largest measuring 45 × 67 × 54 mm). Differential diagnoses included hydatid disease, primary peritoneal neoplasms, and metastatic carcinomatosis (Fig. 1). 

**Figure 2:**
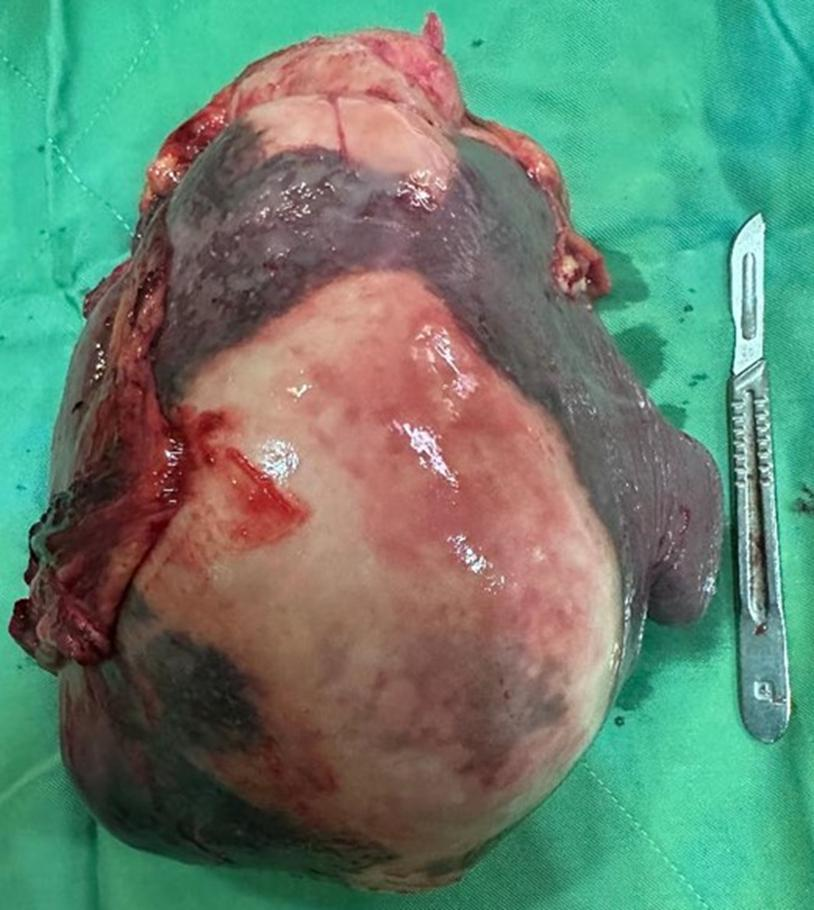
Image of the spleen removed from the patient, which contains several huge cysts.

**Figure 3:**
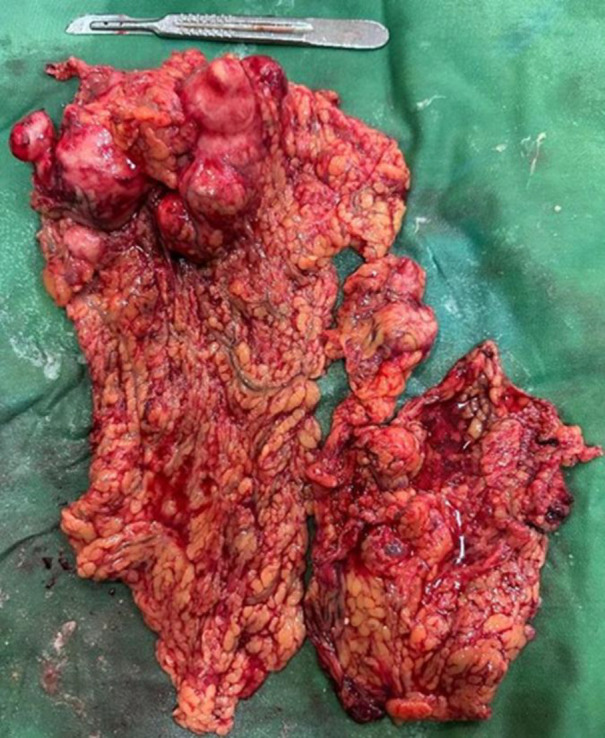
The image of the omentum removed from the patient, which contains a large number of nodular and cystic lesions of different sizes.

**Figure 4:**
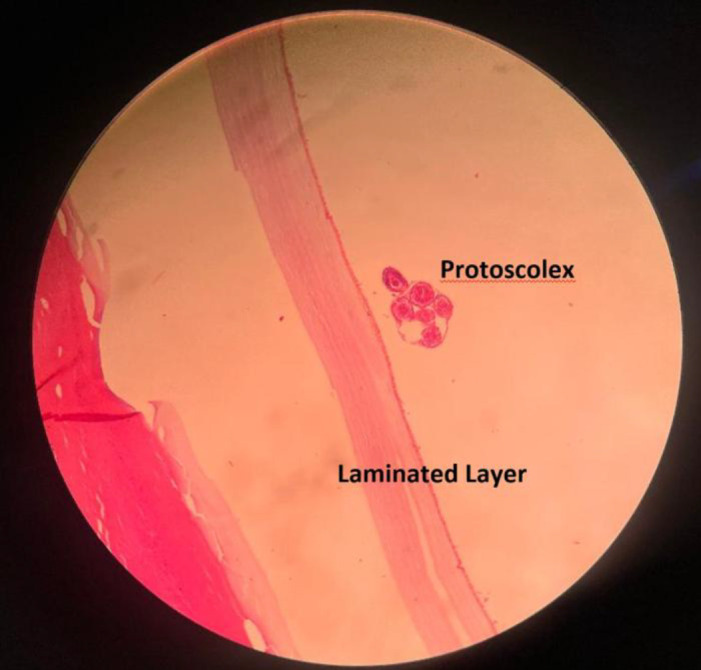
Microscopic view of one of the nodular lesions of the omentum, which shows involvement with hydatid cyst. In this view, the protoscolex is visible next to the laminated layer.

## Discussion

Tapeworms of the *Echinococcus* genus have been recognized as human pathogens since the 1950s. Two major species, *Echinococcus granulosus* and *Echinococcus multilocularis*, are responsible for cystic echinococcosis (CE) and alveolar echinococcosis, respectively. Among these, CE is the more common form. The adult tapeworm resides in the small intestines of carnivores, and its eggs are excreted through feces, contaminating food and water sources. Intermediate hosts, such as herbivores, ingest the eggs through contaminated food. Humans, as accidental intermediate hosts, become infected either through direct contact with infected animals or indirectly via ingestion of contaminated food or water. Once ingested, larvae disseminate through the hematogenous and lymphatic systems (6,7).

Although echinococcosis is globally distributed, endemic regions include Oceania, China, Central Asia, southern and central Russia, the Middle East, Mediterranean countries, parts of Africa, and North America (7). CE is a significant parasitic disease, particularly in low-income and developing regions, where it remains a public health concern due to high morbidity and mortality. According to the World Health Organization, CE is one of 17 neglected tropical diseases. It affects over one million people annually, with approximately 20,000 associated deaths. Disability-adjusted life years (DALYs) attributable to CE exceed 800,000, and global annual treatment costs are estimated at over 3 billion USD (7,8).

CE most commonly affects individuals in their fourth and fifth decades of life and is slightly more prevalent among women. Risk factors include rural residence and frequent contact with livestock or dogs (3). The disease is often asymptomatic and discovered incidentally. While abdominal ultrasound serves as the initial imaging modality, computed tomography (CT) remains the gold standard for diagnosis (9). Serologic tests may assist in diagnosis but are not definitive, as negative results do not exclude CE. Clinical symptoms typically develop years after initial infection and depend on the cyst's size and anatomical location (10). When symptoms are present, dull abdominal pain is the most common complaint. Most CE cases involve a single cyst, typically in the liver, followed by the lungs. Involvement of the central nervous system, muscles, and spleen is rare, and peritoneal or omental involvement is exceedingly uncommon (3).

In the case presented, an elderly man from a rural area with occasional livestock contact presented with nonspecific abdominal pain, weight loss, and a family history of colorectal cancer. Given the presence of red-flag symptoms and iron-deficiency anemia, an initial workup focused on ruling out malignancy. Abdominal ultrasonography revealed cystic lesions in the liver and spleen suggestive of hydatid disease, but serologic testing was negative. CT imaging confirmed the presence of a calcified hepatic cyst and splenomegaly with multiple cystic lesions, along with numerous peritoneal and omental nodules mimicking peritoneal carcinomatosis. Given the clinical uncertainty, diagnostic laparoscopy was performed, which confirmed extensive peritoneal and omental involvement with hydatid disease.

In recent studies, most CE patients have a solitary cyst. In a 2020 study involving 501 CE cases over 15 years, peritoneal involvement was reported in only 1% of patients (3). Peritoneal hydatid cysts typically occur secondary to abdominal trauma or surgery, which can lead to rupture of a hepatic cyst into the abdominal cavity. However, our patient had no history of trauma or surgery. Several hypotheses have been proposed to explain primary peritoneal hydatid disease, including hematogenous or lymphatic spread from a hepatic cyst or rupture of the adventitial layer without rupture of the cyst membrane (4).

The mainstay of treatment includes antiparasitic therapy (typically albendazole 10 mg/kg), surgery, or a combination of both. The primary treatment goal is complete cyst removal, reduction of complications, and prevention of recurrence. Surgical resection remains the most effective method and yields better outcomes when combined with medical therapy. Medical therapy alone may arrest parasitic growth but is generally insufficient for complete cure. It is reserved for patients who are poor surgical candidates (11).

In the present case, splenectomy was performed due to the extensive involvement of the spleen. In addition, all visibly affected omental regions were resected to reduce the risk of cyst rupture, which could lead to peritonitis and anaphylactic shock. Given the patient’s postsplenectomy status and increased susceptibility to encapsulated organisms, he was appropriately vaccinated with the 13-valent pneumococcal conjugate vaccine, with the 23-valent vaccine scheduled later. The calcified liver cyst was not surgically addressed and remained stable, and the patient was treated with oral albendazole for three months. 

## Conclusion

This case highlights that although hydatid cysts of the peritoneum and omentum are rare, they may occur in conjunction with cysts in more common sites such as the liver and spleen. While most peritoneal or omental hydatid cysts result from previous abdominal trauma or surgical manipulation, alternative mechanisms—including hematogenous or lymphatic spread or rupture of the cyst adventitia—should also be considered.

Computed tomography remains the most effective imaging modality for diagnosing hydatid cysts; however, peritoneal and omental cysts may mimic malignant lesions or peritoneal carcinomatosis, leading to diagnostic uncertainty and potential delays in treatment. This case underscores the importance of timely diagnosis, as untreated hydatid cysts carry the risk of rupture, secondary peritonitis, anaphylactic reactions, and increased mortality.

Surgical excision, complemented by postoperative anthelmintic therapy, remains the cornerstone of treatment and is critical in preventing complications and improving patient outcomes.

## Data Availability

Data is available upon reasonable request from the corresponding author.
